# Supplementary material for: Direct detection of molecular intermediates from first-passage times
Source: Sci Adv. 2020 May 1;6(18):eaaz4642. doi: 10.1126/sciadv.aaz4642 (PMC7195145; doi:10.1126/sciadv.aaz4642)
Supplement: aaz4642_SM.pdf [file aaz4642_SM.pdf]

Science Advances
AAAS

advances.sciencemag.org/cgi/content/full/6/18/eaaz4642/DC1

Supplementary Materials for

Direct detection of molecular intermediates from first-passage times

Alice L. Thorneywork*, Jannes Gladrow, Yujia Qing, Marc Rico-Pasto, Felix Ritort, Hagan Bayley, Anatoly B. Kolomeisky, Ulrich F. Keyser

*Corresponding author. Email: at775@cam.ac.uk

Published 1 May 2020, *Sci. Adv.* **6**, eaaz4642 (2020)
DOI: 10.1126/sciadv.aaz4642

This PDF file includes:

Sections S1 to S5
Figs. S1 to S3
References

Supplementary Material

Section S1. Theoretical Background

To start with the simplest case, consider a 1D network of discrete states $i = 0, ...B$, where at $t = 0$ a system starts at some state in the network with $i = A$. Over time, the system can transition between states by moving one step forward in the network to state $i = A + 1$ or by moving one step backwards to state $i = A - 1$. Forward transitions occur with a rate u_A and backward transitions at a rate w_A. We wish to obtain the first passage probability distribution $P_{B,A}(t)$ that describes the probability that a system starting at state A will reach state B at time t without first hitting state 0.

By considering the possible transitions between states, the temporal evolution of the probability distribution can be described by backward master equations as

$$\frac{\partial P_{B,A}(t)}{\partial t} = w_A P_{B,A+1}(t) + u_A P_{B,A-1}(t) - (u_A + w_A) P_{B,A}(t) \tag{1}$$

with boundary conditions

$$P_{B,0}(t) = 0, \text{ for all } t, \tag{2}$$

$$P_{B,B}(t) = \delta(t), \tag{3}$$

$$P_{B,A}(0) = 0, \text{ for } 1 \leq A \leq B-1 \tag{4}$$

To solve this equation a Laplace transform, defined as

$$\tilde{P}_{B,A}(s) = \int_0^\infty \exp(-st) P_{B,A}(t) dt \tag{5}$$

is performed, allowing for Eq. 1 to be written as

$$\tilde{P}_{B,A}(s) = w_A \tilde{P}_{B,A+1}(t) + u_A \tilde{P}_{B,A-1}(s) - (u_A + w_A)\tilde{P}_{B,A}(s) \tag{6}$$

From here, precise details of the solution depend upon the nature of the system (*24*), for example, whether the rates of transition between states are all the same or vary depending upon which state the system is in. However, in all cases, irrespective of these details, a final expression of the form

$$P_{B,A}(t) \propto t^{B-A-1} \tag{7}$$

is obtained. At early times this term dominates, leading to, on a log-log scale, the key result of

$$\ln P_{B,A}(t) \simeq (B - A - 1) \ln t + C \tag{8}$$

corresponding to Eq. 1 of the main text. Physically, the implication of this expression is that, at early times, only the fastest events are observed in which the system moves sequentially from

the initial state to the final state via every intermediate state. In this regime, the probability of transitioning from one intermediate state to the next intermediate state is proportional to the time. For the pathway with $m = B - A - 1$ intermediate states, this will lead to the power-law dependence as described above.

Now consider a general system that involves multiple discrete states. Processes taking place in this system can be viewed as a multi-dimensional network of transitions between these different states. Here, for a process of interest in which a system moves from state A to state B many pathways may connect the initial and final states. As such, in analyzing events that start at the state A and finish at B, it is clear that events corresponding to all of these pathways will be included. However, the time to travel along each possible pathway in the network of discrete states is not the same. In considering short times, only events along the shortest pathway i.e. with the fewest number of intermediate states, will be observed because they will be the fastest. This means that at short times we are probing effectively a one-dimensional part of the system, corresponding to the shortest pathway. While the exact range of times over which these shortest events occur will depend on transition rates it can be shown that this short-time regime will always exist (*34*). As such, the method can also be successfully used for analysis of complex multi-dimensional systems.

Section S2. DNA hairpin sequences

Details of the fabrication of DNA hairpin structures have previously been reported (*32, 33, 36, 37*), including the three state structure used in this work (*32*). The four state hairpin is built from two strands. The first has the following sequence:

AATAGAGACACATATATAATAGATC

TTCGCACTGACTGACGAGCATCACA

AAAATCGACGCTCAAGTCAGAGGTG

GCGAAACCCGACAGGACTATAAAGA

TACCAGGCGTTTCCCCCTGGAAGCT

CCCTCGTGCGCTCTCCTGTTCCGAC

TGTGGTGTAGTCTTGGCAGGAGCTA

AACACGAAACGGGAATCACTTGTAC

CGGCTATCCGAGATGTTGACCTTCG

ACAGAAATTGGCCCTCATAACCCCC

ATGAAAAGCGCATTAAGCCTAGAGG

CGTAAGAACTGGACGTTTGATCGCG

GTATATTGCTGAGTATGCACGTCAC

TTAGTAACTAACATGATAGTTAC + loop (TTTT)

The second strand is the complementary strand to this sequence.

Particle trajectories are acquired from videos using standard image analysis techniques (*38*). As datasets are acquired via an automated process using the optical tweezers, large datasets of trajectories are obtained for each potential landscape (500-4000 trajectories corresponding to more than 10^5 particle positions) (*28, 39*).

Section S3. Analysing particle trajectories and establishing the slope of the linear regime

To calculate the first-passage time distributions in our colloidal system, data is first split into subsets. Each subset corresponds to the particle starting from a different potential minimum, and thus position within the channel. The value of m iattached to each distribution indicates the lowest number of minima that must be crossed by the particle to exit from the channel, from its initial position (a minima in the channel) to its final position (the left or right reservoir).

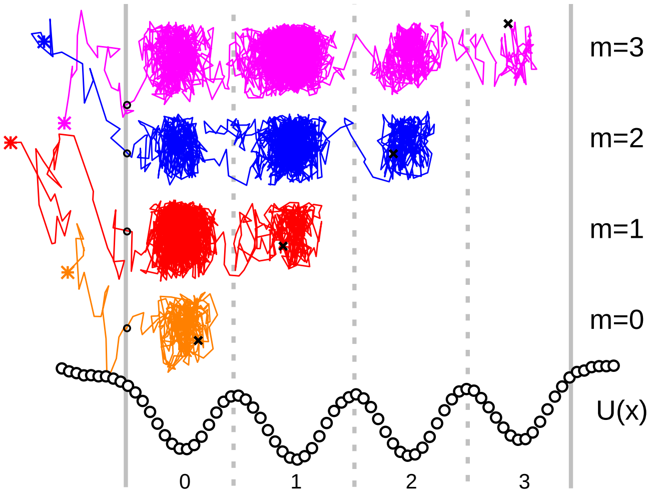

Figure S1: **Typical trajectories contributing to first-passage time distributions**. Trajectories shown contribute to $m = 0, 1, 2, 3$ in a colloidal system with imposed potential minima of depth $\Delta U \sim 3\,k_{\mathrm{B}}T$. Also shown is the corresponding potential landscape $U(x)$. Dashed lines indicate the boundaries between minima and solid grey lines the left and right channel exits. For each trajectory the starting position is marked as a black cross and the final position by coloured stars.

Typical particle trajectories, as used to calculate the distributions shown in Fig. 2A and B in the main manuscript, are shown in Fig. S1. Here, all trajectories correspond to escape to the left reservoir. The corresponding potential landscape, $U(x)$, for the system is also shown, with potential minima depth $\Delta U \sim 3\,k_{\mathrm{B}}T$. Black crosses indicate the starting position of the particle in each trajectory, coloured stars the final position and black circles the position of the particle at which it crosses the left boundary, which is used to determine the first-passage time. Here, $m = 0$ corresponds to crossing 0 minima or one boundary to move from the initial state (minima 0) to final state (left reservoir), $m = 1$ to crossing 1 minima or two boundaries from the initial state (minima 1) to final state (left reservoir), $m = 2$ to crossing 2 minima or three boundaries from the initial state (minima 2) to final state (left reservoir) and $m = 3$ to crossing 3 minima or two boundaries from the initial state (minima 3) to final state (left reservoir).

The scaling behaviour of the short-time regime is determined simply by inspection of the

distributions. As a first step, the distribution for a particular imposed potential is compared to that with no imposed potential (corresponding to free diffusion for the colloidal system as shown in section S4). This allows for the change in shape of the distribution to a more linear behaviour caused by the presence of the potential minima to be identified. Having thereby identified the linear regime of the distribution, we compare this region of the data on a log-log scale to lines of integer slope to determine the scaling that best describes it.

Section S4. First-passage time distributions with no imposed potential landscape

Fig. S2A and B show the first-passage time distributions, $P(t_{\mathrm{FPT}})$, for a system with no imposed potential, where the particle is allowed to diffuse freely before exiting the channel. Here, the distributions have been built from subsets of trajectories that start from different regions of the channel. More specifically, subsets correspond to particles starting from each of four equal regions in x, with $m = 1, 2, 3$, now indicating exit of the particle by crossing at least 1, 2 or 3 regions respectively. These distributions act as a control against which measurements with imposed potential minima can be directly compared. In Fig. S2 the distributions with no imposed potential landscape on both a (A) linear and (B) log-log scale exhibit the expected qualitative behavior for escape from the channel from regions at increasingly large distances from the exit, namely a broadening and a shift in the peak to larger times. On a linear scale the distribution with no-imposed potential landscape looks qualitatively very similar to the behaviour of the first-passage time distributions with an imposed potential landscape (shown for comparison in Fig. S2C). On a log-log scale, however, the distributions with no potential landscape in (B) do not show the linear behaviour at short-times – corresponding to the short-time power law scaling of Eq. 1– that is evident in the short-time regime of the distributions with imposed potential minima in (D). The short-time behaviour of the distributions with no imposed potential is in

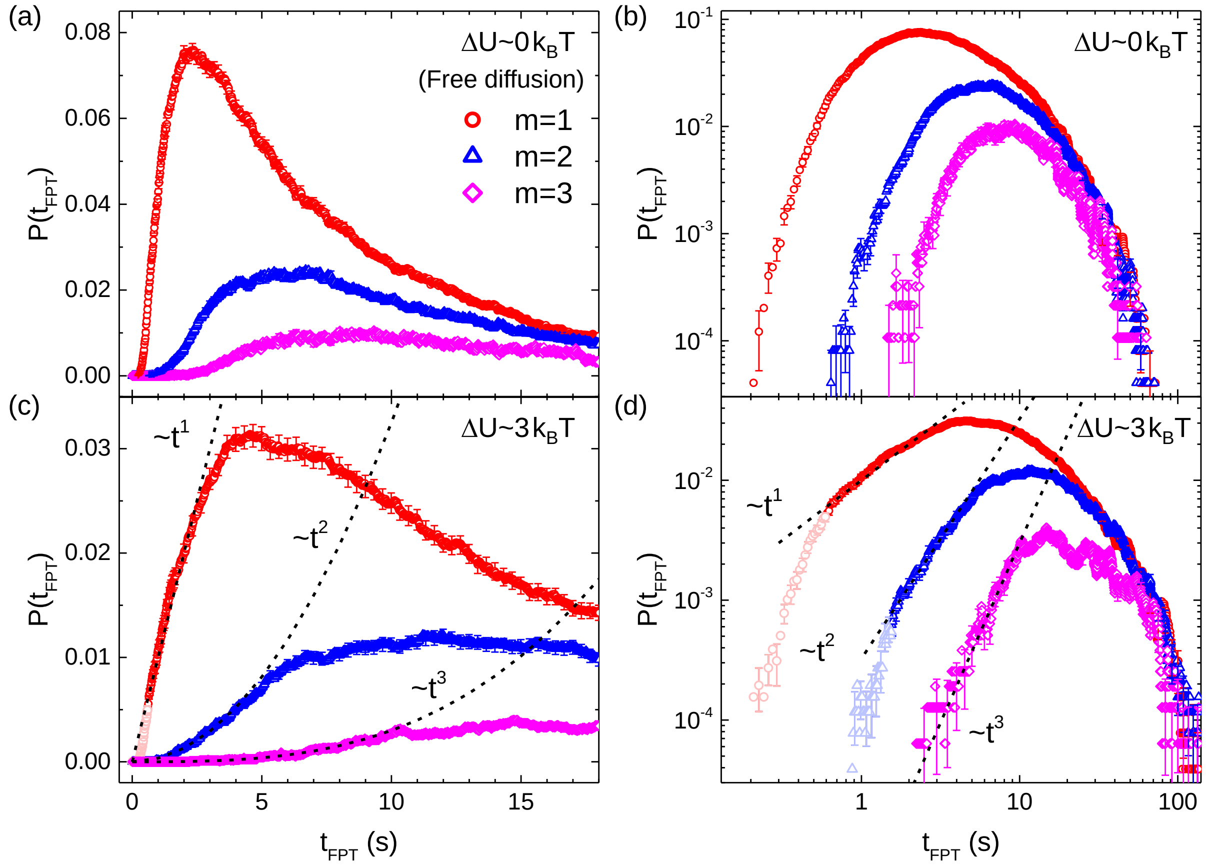

Figure S2: **First-passage time distributions of colloidal particles with and without an imposed potential landscape.** Distributions in (**A**) and (**B**) show $P(t_{\mathrm{FPT}})$ on a linear and log-log scale for freely diffusing particles i.e. with no imposed potential landscape. Dashed lines in B show the theoretical prediction for the first-passage time distribution of a particle in a system with no imposed potential landscape. For comparison, distributions in (**C**) and (**D**) show $P(t_{\mathrm{FPT}})$ on a linear and log-log scale for particles diffusing over a potential landscape composed of four potential minima with depth $\Delta U \sim 3k_{\mathrm{B}}T$ (from main manuscript Fig. 2A and B). Dashed lines indicate the predicted power-law scaling according to Eq. 1 and the legend in panel A applies to all panels.

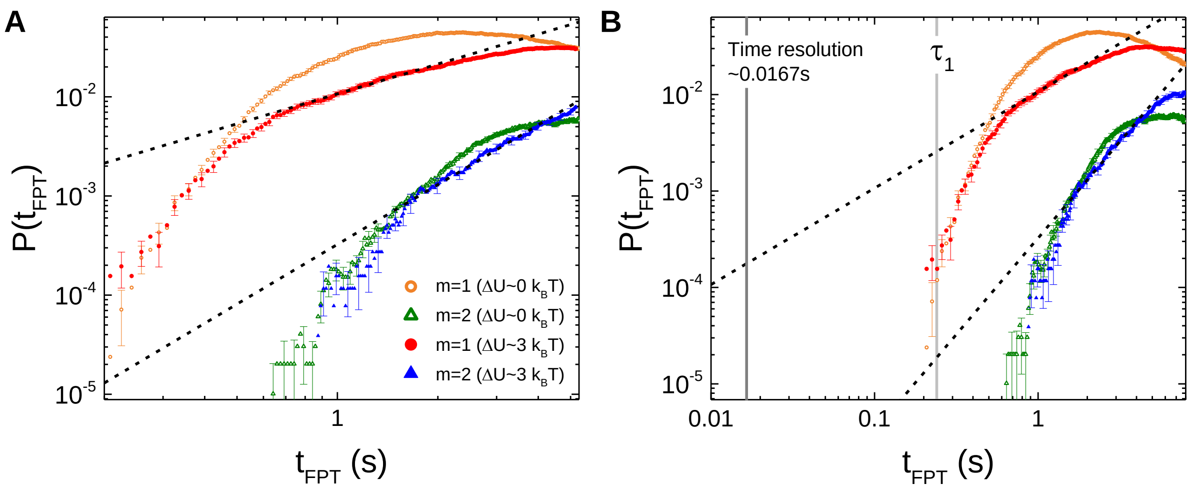

Figure S3: **The very short-time regime of the first-passage time distribution.** (**A**) Comparison of the very short time regime of the first-passage time distributions with no imposed potential and with $\Delta U \sim 3k_{\rm B}T$. Data is shown separately in Fig. S2. Dashed lines indicate the predicted power-law scaling according to main manuscript Eq. 1 as shown in Fig. S2D. (**B**) The same data over a larger range of times. Vertical lines indicate the experimental time resolution and an estimate of the minimum escape time for the system.

fact in good agreement with theoretical predictions for the first-passage time distribution of a particle undergoing free diffusion, shown in Fig. S2B as black dashed lines. These curves are calculated as a numerical solution of the Fokker-Planck equation with the experimental diffusion coefficient, channel length and measured probability distribution of starting positions as input. Importantly, the agreement with the expected theoretical behaviour further emphasises the absence of a linear regime in the data with no imposed potential.

To further highlight the different behaviour seen with and without an imposed potential more explicitly, Fig. S3A shows a direct comparison of the short time regime of the first-passage time distributions shown in Fig. S2. Here, black dashed lines indicate the predicted power-law scaling according to Eq. 1 and the difference in qualitative behaviour of the distributions at short-times can clearly be seen. Fig. S3A also allows for the more similar behaviour of the distributions at very short times to be considered. To achieve this, data for $\Delta U \sim 0\ k_{\rm B}T$ has

been renormalised (leading to a shift downwards) to overlap with the data for $\Delta U \sim 3\ k_{\mathrm{B}}T$. This allows for the shape of the distributions in this limit to be more easily compared. Fig. S3A clearly shows that at very short times i.e. for times at which there is a deviation from the power law scaling for the data with imposed potential landscape, the shape of the distributions with an imposed potential landscape coincides with that of the distributions for the free diffusion case. This suggests that these very short time events correspond to effectively 'ballistic' motion in the direction of the exit with particles moving so rapidly across the potential landscape that they do not have enough time to feel the potential minima.

Furthermore the very short time deviations highlight the approximate nature of the modeling of our experimental system, which has a continuous potential landscape, in terms of a Markov Jump process, as in the theoretical description of movement through a discrete network. This assumption is only valid in the limit in which there is separation of timescales such that the time spent within a minimum is much longer than the time spent moving between the minima and thus is only valid for sufficiently deep minima. Indeed, alternative theoretical approaches that more explicitly consider continuous diffusion processes find different scaling behaviour at short-times (as discussed in (*40–42*)) and so we note our observation of these very short time deviations may reflect the different behaviour associated with a continuous landscape.

In Fig. S3B we show the same data but now over a wider range of times. Here we also indicate as vertical lines two key timescales for the system. The first is the experimental time resolution of $\sim 0.0167s$ for the frame rate of 60 fps. It is clear from Fig. S3B that the time resolution is around an order of magnitude smaller than the shortest events. This supports our conclusion that the deviations from the linear scaling at short times do not arise from events that are missed due to the finite time resolution.

Indicating the time resolution also clearly shows that there is an initial period of time for which no first passage events occur. This is because the particle must diffuse a certain distance

to the exit before it can escape, and the length of time associated with this diffusion corresponds to the region between the two vertical lines in Fig. S3B. Here, the second vertical line, marked τ_1, shows a theoretical estimate of the shortest time at which it is probable to observe an event. To estimate τ_1, we first obtain the 1D probability distribution of particle displacements, $P(\Delta x, t)$, for a particle with the diffusion coefficient measured in our experiments. Multiplying this probability distribution by the number of experimental trajectories and then integrating the area under the distribution with $\Delta x > L/4$, where L is the channel length, provides an estimate of the number of particles that would display this displacement after time t. As the value of t increases, the probability of finding a particle at a larger distance from its initial position increases, leading to a broader distribution. The value of τ_1 corresponds to the time required for the distribution to have become sufficiently broad that this integral is approximately equal to 1, i.e. for there to be an appreciable probability that a particle will diffuse far enough to escape. This estimate is in good agreement with the shortest events observed in our experiment.

Section S5. Linking the length of the linear regime to minima depth

In Fig. 2D of the main manuscript we observe a linear increase in Δt, the length of the power-law scaling regime of $P(t_{FPT})$, with $\exp(\Delta U/k_{\mathrm{B}}T)$. Furthermore, the gradient of Δt against $\exp(\Delta U/k_{\mathrm{B}}T)$ increases with m. Note that we do not include data for $m = 3$ at $\Delta U \sim 5k_BT$ as the very long times associated with this distribution mean we have insufficient statistics to accurately estimate the short-time regime.

To rationalise this we consider the main features of the theoretical model used to derive Eqn. 1 from the main manuscript (*24*). The theoretical model follows Kramer's theory, which assumes that transitions between the states in the network involve only barrier-crossing, with barriers that are large comparable to $k_{\mathrm{B}}T$. The short-time regime of the distribution reflects

movement of the particle along the single shortest pathway, i.e. directly towards the exit crossing intermediate barriers in only one direction. The time required to follow this shortest path will therefore be related to the number of barriers crossed multiplied by the time necessary to cross a barrier. The time for barrier crossing is proportional to $\exp(\Delta U/k_{\mathrm{B}}T)$. As such it is clear that Δt should scale with $\exp(\Delta U/k_{\mathrm{B}}T)$. Furthermore, as the number of minima that must be crossed increases, the time to follow the shortest path will also increase, rationalising the increase in gradient in Fig. 2D with increasing value of m.

Having established the dependence of the length of the linear regime on the potential minima depth, this relationship can be used to determine the effective value of $\Delta U/k_{\mathrm{B}}T$ in the molecular hopper system. To compare the length of the linear regime in different systems, however, the differing typical time and lengthscales in each system must be taken into account. To account for this the plot of Δt against $\exp(\Delta U/k_{\mathrm{B}}T)$ in Fig. 2D is expressed as:

$$\Delta t \sim C_m t_0 \exp(\Delta U/k_{\mathrm{B}}T) \tag{9}$$

where C_m is a constant, dependent on m, and t_0 is the time to move across a state in the absence of a potential minima. This can be rearranged as

$$\frac{\Delta U}{k_{\mathrm{B}}T} \sim \ln\left(\frac{\Delta t}{t_0 C_m}\right). \tag{10}$$

For the colloidal system, the $m = 1$ line in Fig. 2D has a gradient $C_m t_0 = 0.0658$. The typical diffusion coefficient of a colloidal particle in the channel is approximately $0.25\ \mu\mathrm{m}^2\mathrm{s}^{-1}$ and the distance between the potential minima is 1.2 μm. As such, the typical timescale for moving between states in the absence of an imposed potential landscape, $t_0 \sim 3$ s. This allows for the constant $C_m = 0.022$ for the $m = 1$ process to be obtained.

For the nanoscale molecular hopper system, the distance between states is approximately 0.68 nm and the typical timescale to move between states in the absence of footholds is approximately 5 μs (*43*). Furthermore, in the m=1 distribution for the hopper (main manuscript

Fig. 3B), the length of the linear regime, $\Delta t = 2.5$s. Substituting these values for Δt, t_0 and C_m into Eq. (2) allows ΔU to be calculated for the molecular hopper as approximately 17 $k_\mathrm{B}T$.

REFERENCES AND NOTES

1. J. H. Espenson, *Chemical Kinetics and Reaction Mechanism* (McGraw-Hill Companies, ed. 2, 2002).

2. P. L. Houston, *Chemical Kinetics And Reaction Dynamics* (Courier Corporation, 2012).

3. H. S. Chung, K. McHale, J. M. Louis, W. A. Eaton, Single-molecule fluorescence experiments determine protein folding transition path times. *Science* **335**, 981–984 (2012).

4. K. Neupane, D. A. N. Foster, D. R. Dee, H. Yu, F. Wang, M. T. Woodside, Direct observation of transition paths during the folding of proteins and nucleic acids. *Science* **352**, 239–242 (2016).

5. M. A. Micheelsen, C. Rischel, J. Ferkinghoff-Borg, R. Guerois, L. Serrano, Mean first-passage time analysis reveals rate-limiting steps, parallel pathways and dead ends in a simple model of protein folding. *Europhys. Lett*. **61**, 561–566 (2003).

6. P. G. Wolynes, J. N. Onuchic, D. Thirumalai, Navigating the folding routes. *Science* **267**, 1619–1620 (1995).

7. M. Karplus, Behind the folding funnel diagram. *Nat. Chem. Biol.* **7**, 401–404 (2011).

8. K. A. Dill, J. L. MacCallum, The protein-folding problem, 50 years on. *Science* **338**, 1042–1046 (2012).

9. T. Chou, Kinetics and thermodynamics across single-file pores: Solute permeability and rectified osmosis. *J. Chem. Phys.* **110**, 606–615 (1999).

10. A. M. Berezhkovskii, M. A. Pustovoit, S. M. Bezrukov, Channel-facilitated membrane transport: Transit probability and interaction with the channel. *J. Chem. Phys.* **116**, 9952–9956 (2002).

11. W. R. Bauer, W. Nadler, Thermodynamics of competitive molecular channel transport: Application to artificial nuclear pores. *PLOS ONE* **5**, e15160 (2010).

12. A. Zilman, Effects of multiple occupancy and interparticle interactions on selective transport through narrow channels: Theory versus experiment. *Biophys. J*. **96**, 1235–1248 (2009).

13. S. Agah, M. Pasquali, A. B. Kolomeisky, Theoretical analysis of selectivity mechanisms in molecular transport through channels and nanopores. *J. Chem. Phys*. **142**, 044705 (2015).

14. A. B. Kolomeisky, Channel-facilitated molecular transport across membranes: Attraction, repulsion, and asymmetry. *Phys. Rev. Lett.* **98,** 048105 (2007).

15. S. Singh, P. Menczel, D. S. Golubev, I. M. Khaymovich, J. T. Peltonen, C. Flindt, K. Saito, É. Roldán, J. P. Pekola, Universal first-passage-time distribution of non-gaussian currents. *Phys. Rev. Lett.* **122**, 230602 (2019).

16. J.-P. Bouchaud, R. Cont, A Langevin approach to stock market fluctuations and crashes. *Eur. Phys. J. B.* **6**, 543–550 (1998).

17. R. Chicheportiche, J.-P. Bouchaud, in *First-passage Phenomena and their Applications*, R. Metzler, S. Redner, G. Oshanin, Eds. (World Scientific, 2014), chap. 18, p. 447.

18. J. Jurczyk, T. Rehberg, A. Eckrot, I. Morgenstern, Measuring critical transitions in financial markets. *Sci. Rep*. **7**, 11564 (2017).

19. H. Yu, A. N. Gupta, X. Liu, K. Neupane, A. M. Brigley, I. Sosova, M. T. Woodside, Energy landscape analysis of native folding of the prion protein yields the diffusion constant, transition path time, and rates. *Proc. Natl. Acad. Sci. U.S.A*. **109**, 14452–14457 (2012).

20. H. S. Chung, W. A. Eaton, Protein folding transition path times from single molecule FRET. *Curr. Opin. Struc. Biol*. **48**, 30–39 (2018).

21. N. Q. Hoffer, M. T. Woodside, Probing microscopic conformational dynamics in folding reactions by measuring transition paths. *Curr. Opin. Chem. Biol*. **53**, 68–74 (2019).

22. D. E. Makarov, *Single Molecule Science: Physical Principles and Models* (CRC Press, 2015).

23. D. J. Wales, Exploring energy landscapes. *Annu. Rev. Phys. Chem.* **69**, 401–425 (2018).

24. X. Li, A. B. Kolomeisky, Mechanisms and topology determination of complex chemical and biological network systems from first-passage theoretical approach. *J. Chem. Phys.* **139**, 144106 (2013).

25. Q. H. Wei, C. Bechinger, P. Leiderer, Single-file diffusion of colloids in one-dimensional channels. *Science* **287**, 625–627 (2000).

26. E. Wonder, B. Lin, S. A. Rice, Single-particle diffusion in dense inhomogeneous colloid suspensions in ribbon channels. *Phys. Rev. E* **84**, 041403 (2011).

27. X. Yang, C. Liu, Y. Li, F. Marchesoni, P. Hänggi, H. P. Zhang, Hydrodynamic and entropic effects on colloidal diffusion in corrugated channels. *Proc. Natl. Acad. Sci. U.S.A.* **114**, 9564–9569 (2017).

28. J. Gladrow, M. Ribezzi-Crivellari, F. Ritort, U. F. Keyser, Experimental evidence of symmetry breaking of transition-path times. *Nat. Commun*. **10**, 55 (2019).

29. R. D. L. Hanes, C. Dalle-Ferrier, M. Schmiedeberg, M. C. Jenkins, S. U. Egelhaaf, Colloids in one dimensional random energy landscapes. *Soft Matter* **8**, 2714–2723 (2012).

30. M. P. N. Juniper, A. V. Straube, R. Besseling, D. G. A. L. Aarts, R. P. A. Dullens, Microscopic dynamics of synchronization in driven colloids. *Nat. Commun.* **6**, 7187 (2015).

31. Y. Qing, S. A. Ionescu, G. S. Pulcu, H. Bayley, Directional control of a processive molecular hopper. *Science* **361**, 908–912 (2018).

32. N. Forns, S. D. Lorenzo, M. Manosas, K. Hayashi, J. M. Huguet, F. Ritort, Improving signal/noise resolution in single-molecule experiments using molecular constructs with short handles. *Biophys. J.* **100**, 1765–1774 (2011).

33. J. M. Huguet, C. V. Bizarro, N. Forns, S. B. Smith, C. Bustamante, F. Ritort, Single molecule derivation of salt dependent base-pair free energies in DNA. *Proc. Natl. Acad. Sci. U.S.A.* **107**, 15431–15436 (2010).

34. X. Li, A. B. Kolomeisky, A. Valleriani, Pathway structure determination in complex stochastic networks with non-exponential dwell times. *J. Chem. Phys.* **140**, 184102 (2014).

35. A. Valleriani, X. Li, A. B. Kolomeisky, Unveiling the hidden structure of complex stochastic biochemical networks. *J. Chem. Phys.* **140**, 064101 (2014).

36. J. M. Huguet, N. Forns, F. Ritort, Statistical properties of metastable intermediates in DNA unzipping. *Phys. Rev. Lett.* **103**, 248106 (2009).

37. M. Rico-Pasto, I. Pastor, F. Ritort, Force feedback effects on single molecule hopping and pulling experiments. *J. Chem. Phys.* **148**, 123327 (2018).

38. J. Crocker, D. Grier, Methods of digital video microscopy for colloidal studies. *J. Colloid Interface Sci*. **179**, 298–310 (1996).

39. Y. Tan, J. Gladrow, U. F. Keyser, L. Dagdug, S. Pagliara, Particle transport across a channel via an oscillating potential. *Phys. Rev. E* **96**, 052401 (2017).

40. A. Godec, R. Metzler, Universal proximity effect in target search kinetics in the few- encounter limit. *Phys. Rev. X* **6**, 041037 (2016).

41. D. Hartich, A. Godec, Duality between relaxation and first passage in reversible Markov dynamics: Rugged energy landscapes disentangled. *New J. Phys.* **20**, 112002 (2018).

42. D. Hartich, A. Godec, Interlacing relaxation and first-passage phenomena in reversible discrete and continuous space Markovian dynamics. *J. Stat. Mech.: Theory Exp* **2019**, 034002 (2019).

43. G. Maglia, M. R. Restrepo, E. Mikhailova, H. Bayley, Enhanced translocation of single DNA molecules through alpha-hemolysin nanopores by manipulation of internal charge. *Proc. Natl. Acad. Sci. U.S.A.* **105**, 19720–19725 (2008).
